# Self-Care Program as a Tool for Alleviating Anxiety and Loneliness and Promoting Satisfaction With Life in High School Students and Staff: Randomized Survey Study

**DOI:** 10.2196/56355

**Published:** 2024-09-30

**Authors:** Priya Iyer, Lina Iyer, Nicole Carter, Ranjani Iyer, Amy Stirling, Lakshmi Priya, Ushma Sriraman

**Affiliations:** 1 Department of Computer Science University of Michigan Ann Arbor, MI United States; 2 Novi High School Novi, MI United States; 3 Department of Education Heartfulness Institute San Ramon, CA United States; 4 Michigan Online School Gobles, MI United States; 5 Department of Humanities Lalaji Memorial Omega International School Chennai India

**Keywords:** Heartfulness, anxiety, loneliness, high school, satisfaction with life, self-care, develop, stress, stress management, effectiveness, life satisfaction, students, student, support, web-based program, time management, educational, mental health, tool, tools

## Abstract

**Background:**

The COVID-19 global pandemic has led to a marked increase in anxiety levels, significantly affecting the well-being of individuals worldwide. In response to this growing concern, interventions aimed at enhancing social-emotional skills and promoting mental health are more crucial than ever.

**Objective:**

This global study aimed to examine the effectiveness of a self-care program on anxiety, loneliness, and satisfaction with life in high school students and staff in a randomized, waitlist control trial with baseline and postintervention assessments.

**Methods:**

The 4-week web-based self-care program, offered by the Heartfulness Institute, is designed to develop social-emotional skills through stress management and self-observation. The web-based program was a positive intervention that offered support to the students and staff to build specific skills, such as reflection, observation, positivity, time management, and goal setting. In this study, the sample consisted of a total of 203 high school students and staff randomized into a control waitlisted group (students: n=57 and staff: n=45) and a Heartfulness group (students: n=57 and staff: n=44) from 3 schools. Both the groups completed web-based surveys at weeks 0, 4, and 8, assessing their anxiety, loneliness, and satisfaction with life scores using Generalized Anxiety Disorder-7 Scale (GAD-7 and Severity Measure for Generalized Anxiety Disorder—Child Age 11-17), Satisfaction With Life scale (SWLS) and Satisfaction With Life Scale-Child (SWLS-C), and the University of California, Los Angeles (UCLA) Loneliness Scale. Survey responses were each individually analyzed using repeated measures ANOVA.

**Results:**

The study received institutional review board approval on February 3, 2022. Participant recruitment lasted from the approval date until March 30, 2022. The 4-week program for the Heartfulness group started on April 4, 2024. There was a significant 3-way interaction among time, group, and school showing a decrease in anxiety and loneliness scores and an increase in satisfaction-with-life scores (*P*<.05). In students in the Heartfulness group, there was strong evidence to suggest a significant mean difference in GAD-7, SWLS, and UCLA scores between week 0 and week 4 at all schools (*P*<.001). In staff in the Heartfulness group, there was strong evidence to suggest a significant mean difference in GAD-7, SWLS, and UCLA scores between week 0 and week 4 at all schools (*P*<.001).

**Conclusions:**

The pandemic brought severe educational and social changes that triggered a decline in mental health in schools. This study showed the effectiveness of noninvasive self-care tools used digitally to significantly decrease anxiety and loneliness scores and increase satisfaction of life scores in the participants.

**Trial Registration:**

ClinicalTrials.gov NCT05874232; https://clinicaltrials.gov/ct2/show/NCT05874232

## Introduction

Individuals affected by an infectious disease outbreak, such as a pandemic, often experience increased anxiety, particularly around contracting the illness, a higher incidence of mental health difficulties, and heightened feelings of helplessness and stigma [1-5]. The circumstances surrounding the COVID-19 pandemic have increased stress and anxiety in people, including high school students [6-8]. Research on the psychological impacts of quarantine found that it was associated with feelings of anxiety, exhaustion, and demotivation, which could persist after the quarantine has ended [9]. Psychological well-being problems have become increasingly common, and the current COVID-19 pandemic has negatively impacted health and well-being with increased anxiety and depression levels [10]. It is likely that satisfaction with life has been adversely affected [11].

Loneliness poses a significant health problem for a sizeable part of the population with increased risks in terms of depression, anxiety, suicidal ideation, health behavior, and health care use [12]. Various studies have documented high levels of loneliness and an increase in loneliness since the start of the pandemic [13-17]. Satisfaction with life is a key component of overall well-being and is influenced by various factors, including mental health, social relationships, and physical health. During the pandemic, the disruption of daily routines, social isolation, and uncertainty about the future have likely contributed to a decrease in life satisfaction among individuals, particularly among adolescents and young adults. Furthermore, research suggests that quarantine might be particularly disruptive for adolescents, who tend to particularly need interaction with peers to support mental well-being [9].

Schools play an essential role in the psychological, social, and academic development of children and employ the largest number of youth mental health providers [18,19]. This is perhaps why approximately 80% of school-aged children with mental and behavioral health needs rely on school-based services [20]. Prolonged isolation can adversely affect physical and emotional health, altering sleep and nutritional rhythms, as well as reducing opportunities for movement [21]. As a result, the natural channels of human expression and pleasure become depressed, with attendant impacts on mood and subjective well-being [22].

One potential intervention to address these challenges is the Heartfulness Self-Care program, a simple yet profound 4-week heart-based program developed by the Heartfulness Institute. This program aims to create a loving and compassionate learning environment to nurture individual well-being. The Heartfulness approach also encourages the building of stress management and social-emotional skills charted by the Collaborative for Academic, Social, and Emotional Learning: self-awareness, self-management, social-awareness, relationship skills, and decision-making skills [23]. The program includes a unique practice of rejuvenation at the end of the day, which may help alleviate the emotional burden of loneliness. Previous research has shown correlations between Heartfulness programs and improvements in stress, anxiety, loneliness, and emotional well-being [24-26].

The aim of this study was to examine the impact of a web-based 4-week Heartfulness Self-Care program on anxiety, satisfaction with life, and loneliness levels in high school students (grades 9-12) and staff. No experimental study has been done to look at these parameters and this study investigated whether using a web-based approach of a heart-based relaxation program led to measurable changes in anxiety, satisfaction with life, and loneliness. The program was offered through a joint initiative of Heartfulness Institute, school 1 in Michigan, school 2 in Michigan, and school 3 in India.

The primary hypothesis of this study is that participation in the Heartfulness Self-Care program will significantly reduce symptoms of anxiety and loneliness while enhancing satisfaction with life among high school students and staff. This study seeks to innovate by integrating heart-based practices with contemporary psychological well-being strategies, offering a novel approach to addressing mental health issues during and after the pandemic. By providing empirical evidence, this study aims to contribute to the growing body of literature on effective mental health interventions and offer actionable insights for educators, mental health professionals, and individuals seeking to improve their psychological well-being.

## Methods

### Design

This quantitative study was a randomized, waitlist control trial with an assessment conducted at baseline (week 0) and postintervention (week 4 and week 8). High school students and staff from 3 schools were randomly split into 2 groups. The first group (Heartfulness) engaged in a Heartfulness Self-Care program for 4 weeks and then engaged in self-practice for weeks 5-8. The second group (waitlist control) followed their daily routine and did no meditation for the first 4 weeks, then participated in the Heartfulness Self-Care program for weeks 5-8.

### Recruitment

Participants were selected using convenience sampling from 3 high schools in Michigan and India. The schools provided 3 different settings and a platform to explore the impact of the web-based program as follows:

- School 1: located in Novi, Michigan, a suburb of Detroit, Michigan, with a population of 2000 students in grades 9-12, and a staff of 125 teachers, 6 counselors, 2 social workers, and 30 additional support staff members. The student body is composed of 43% (860/2000) White (non-Hispanic), 33% (660/2000) Black, 12% (240/2000) Hispanic, and 12% (240/2000) Asian.
- School 2: a fully web-based school in Michigan with an enrollment of 865 students in grades 6-12 as of February 2022, and 63 staff members. The demographic breakdown includes 60.13% (520/865) economically disadvantaged, 15.5% (134/865) special education, 59% (510/865) Caucasian, 28% (242/865) African American, 6% (52/865) Hispanic/Latino, 2% (17/865) Asian, 3% (26/865) American Indian, and 3% (26/865) other.
- School 3: located in India with an enrollment of 6000 students from various countries, including 1112 students in grades 10-12, 98.75% (1098/1112) of whom are Indian and 1.25% (14/1112) foreign nationals. The school employs over 400 teaching staff and more than 100 nonteaching staff.

### Measures

The 3 surveys used the Generalized Anxiety Disorder-7 Scale (GAD-7 and Severity Measure for Generalized Anxiety Disorder—Child Age 11-17), Satisfaction With Life Scale (SWLS), and Satisfaction With Life Scale-Child (SWLS-C), and the University of California, Los Angeles (UCLA) Loneliness Scale have been researched and validated in many studies [27-31]. The GAD-7 is a reliable and valid measure of anxiety severity, with Cronbach α typically exceeding 0.85, indicating high internal consistency. The scale has been extensively validated across different populations and settings, showing strong convergent and discriminant validity. GAD-7 score is calculated by assigning scores of 0, 1, 2, and 3 to the response categories, respectively, of “not at all,” “several days,” “more than half the days,” and “nearly every day.” GAD-7 total score for the 7 items ranges from 0 to 21: 0-4 is minimal anxiety, 5-9 is mild anxiety, 10-14 is moderate anxiety, and 15-21 is severe anxiety. The SWLS is a 7-point Likert-style response scale. The possible range of scores is 5-35, with a score of 20 representing a neutral point on the scale. Scores between 5-9 indicate the respondent is extremely dissatisfied with life, whereas scores between 31-35 indicate the respondent is extremely satisfied. The SWLS has demonstrated high reliability, with Cronbach α values around 0.87 to 0.92, reflecting excellent internal consistency. Its validity has been confirmed through significant correlations with other measures of well-being and mental health, establishing both convergent and concurrent validity. The UCLA Loneliness Scale has shown high reliability with Cronbach α values typically above 0.90, indicating excellent internal consistency. The scale’s validity is well-supported through its strong associations with related constructs such as social support and mental health outcomes, affirming its convergent and criterion validity. The 20-item UCLA Loneliness Scale, version 3, is designed to measure subjective feelings of loneliness and social isolation. The response items scale ranged from 1 (never) to 4 (often). Loneliness sum scores hence ranged from 20 (low) to 80 (high).

Participants filled out the GAD-7, SWLS, and UCLA Loneliness Scale (version 3) before the program at week 0, after 4 weeks, and after 8 weeks.

### Intervention

**Figure 1:**
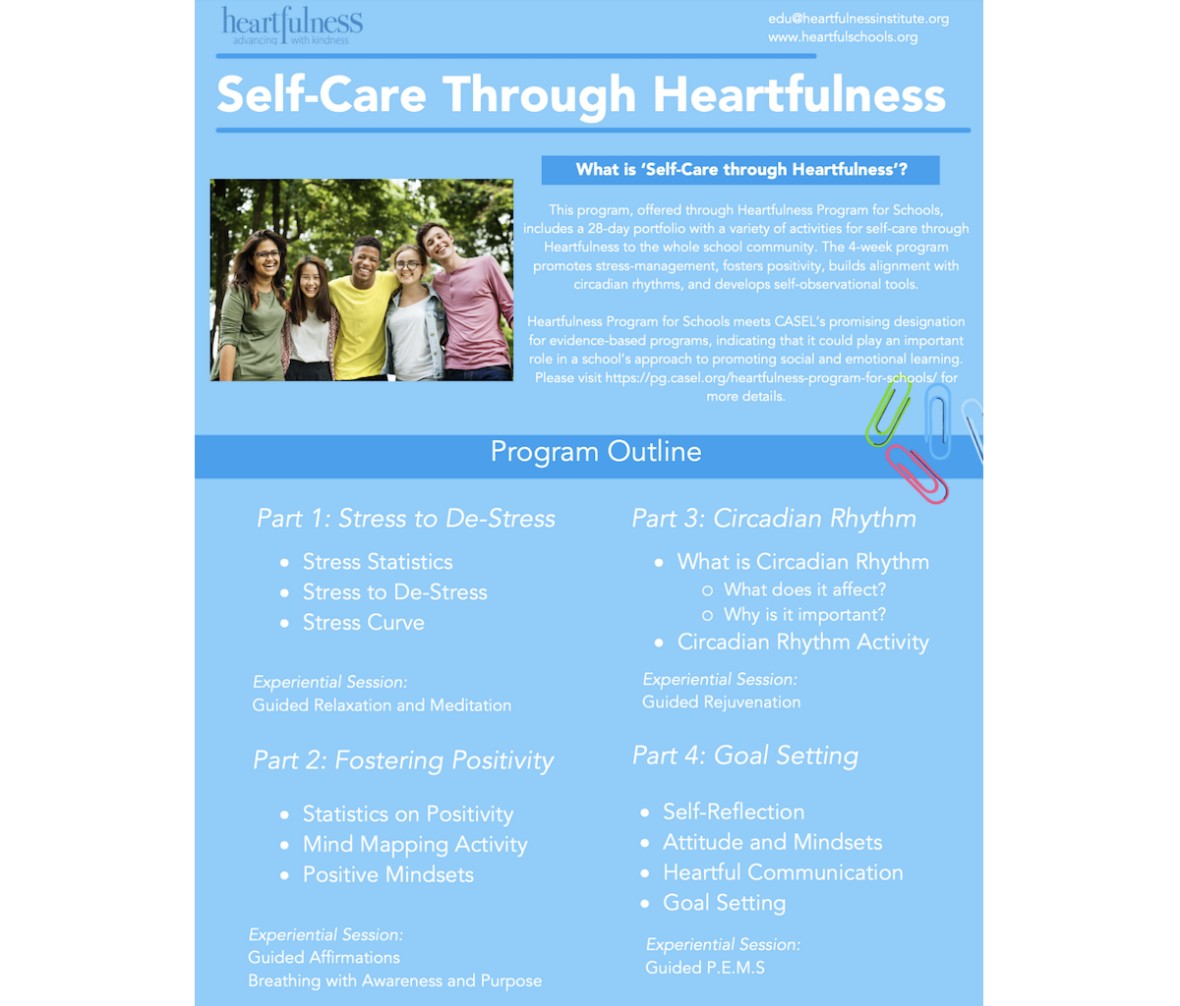
Details of self-care program.

The web-based 4-week self-care program, offered through Heartfulness Program for Schools, included a variety of activities for self-care for the whole school community. Participants received an e-portfolio listing the daily activities and began with a 30-minute webinar each week, that included a hands-on activity and a guided experience of the Heartfulness tool that was practiced for the week. This weekly webinar also focused on step-by-step instructions for daily activities. The 15-minute daily activity for the rest of the week included activities to manage stress, foster positivity, align with the daily circadian rhythm, and set goals with self-observation. The guided tools included an experience to relax, meditate, affirm, breathe, rejuvenate, and self-observe. The program details are available in Figure 1.

Participants who provided consent to participate in the study were randomly picked by a web-based team generator tool to be in group A (Heartfulness group) and group B (control group: waitlist for Heartfulness group) [32]. Group A was sent a 28-day calendar to start their program, while group B worked on their routine and waited for their program to begin later. The students and staff who participated in the self-care program attended four webinars digitally followed by daily activities. As the activities of the study were self-paced, it was suggested that participants who only completed the previous week’s activities could attend the next upcoming webinar. However, individual follow-ups and gentle reminders to complete the webinars, activities, and surveys were given.

### Data Collection

**Figure 2:**
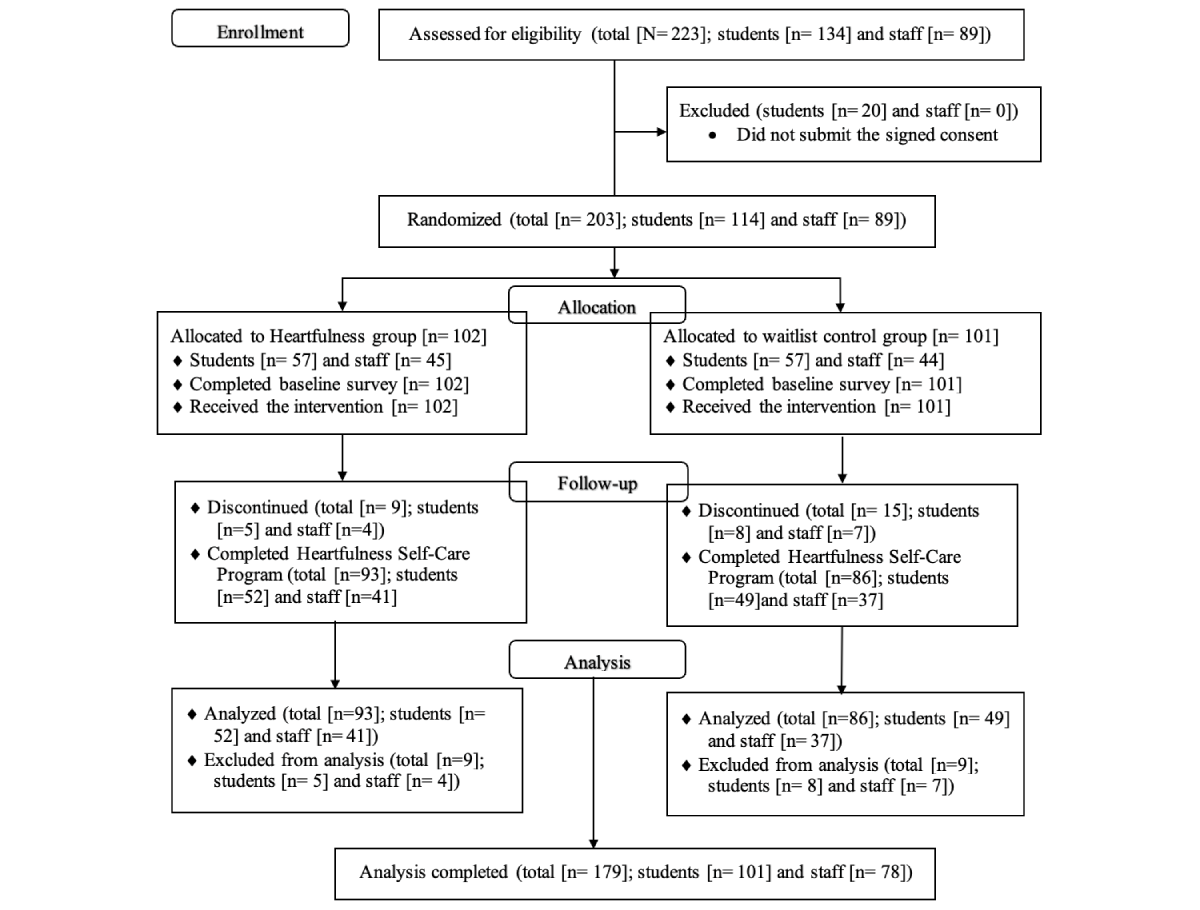
CONSORT (Consolidated Standards of Reporting Trials) flowchart of participants’ enrolment.

This study enrolled 223 consented participants, of which 179 (80.3%) participants completed the study and 44 (19.7%) participants dropped out. After completing the informed consent and baseline survey, using a web-based generator tool participants were randomly placed into the Heartfulness group (n=112; 19 dropped and did not complete the program) and the waitlist control group (n=111; 25 dropped and did not complete the program). Figure 2 shows the study design flowchart in accordance with CONSORT (Consolidated Standards of Reporting Trials) guidelines. Inclusion criteria included high school students aged 14 years or older and staff at the participating schools, while non-English speakers were excluded. The sample size formula ensured sufficient power based on previous studies indicating the program’s impact on loneliness [26].

### Statistical Analysis

Descriptive statistics were used to extract information about characteristics of the sample population from the intake assessment, such as frequency distribution and measures of central tendency.

The survey responses to the anxiety, satisfaction with life, and loneliness surveys were each individually analyzed by repeated measures ANOVA. The repeated measure was time (0, 4, and 8 weeks). The repeated measure accounts for potentially correlated observations as the same participant was measured at 3 time points. Independent variables were included for group (Heartfulness or control) and school type (school 1, school 2, and school 3). A first-order autoregressive covariance structure was found to be the best fit by the Akaike information criterion and was used throughout [33]. Denominator degrees of freedom were calculated through the Kenward-Roger methodology [34]. Model assumptions were assessed through residual plots and deemed met for all 6 repeated measures of ANOVA.

When significant interactions between variables were detected, post hoc multiple comparisons of interest were carried out by the Bonferroni sequentially rejective multiple comparison procedure [35]. Bonferroni’s sequentially rejective multiple comparison procedure cannot calculate confidence intervals, so the usual Bonferroni multiple comparison procedure generated those. The usual Bonferroni procedure is more conservative (ie, results in higher *P* values) than the sequentially rejective version, so it is possible for a statistically significant *P* value for the sequentially rejective version to have a confidence interval that contains 0. The usual Bonferroni procedure also was used for all post hoc comparisons within a single variable. Both procedures ensure that the type I error rate (ie, false positive rate) for each ANOVA is at most α=.05.

### Ethical Considerations

The study was approved by Solutions IRB (Protocol #2022/01/2) through an expedited review process. Participants were recruited between February and March 2022 through flyers in schools and emails to high school students and staff. The inclusion criteria were high school students aged 14 years and older and staff at the 3 schools willing to participate in the study. Exclusion criteria included non–English-speaking high school students. Parental consent was obtained for minors.

## Results

### Participant Demographics

The data showed that out of 101 students, 10% (10/101) were in grade 9, 23% (23/101) in grade 10, 44% (45/101) in grade 11, 23% (23/101), 19% (19/101) were male, 76% (77/101) were female, and 5% (5/101) preferred not to say. Of the 78 staff members who participated, about 71% (55/78) were teachers, and 79% (61/78) were female (Table S1 in Multimedia Appendix 1 and Table S1 in Multimedia Appendix 2 for demographic details).

### Intervention Effects on Students

**Figure 3:**
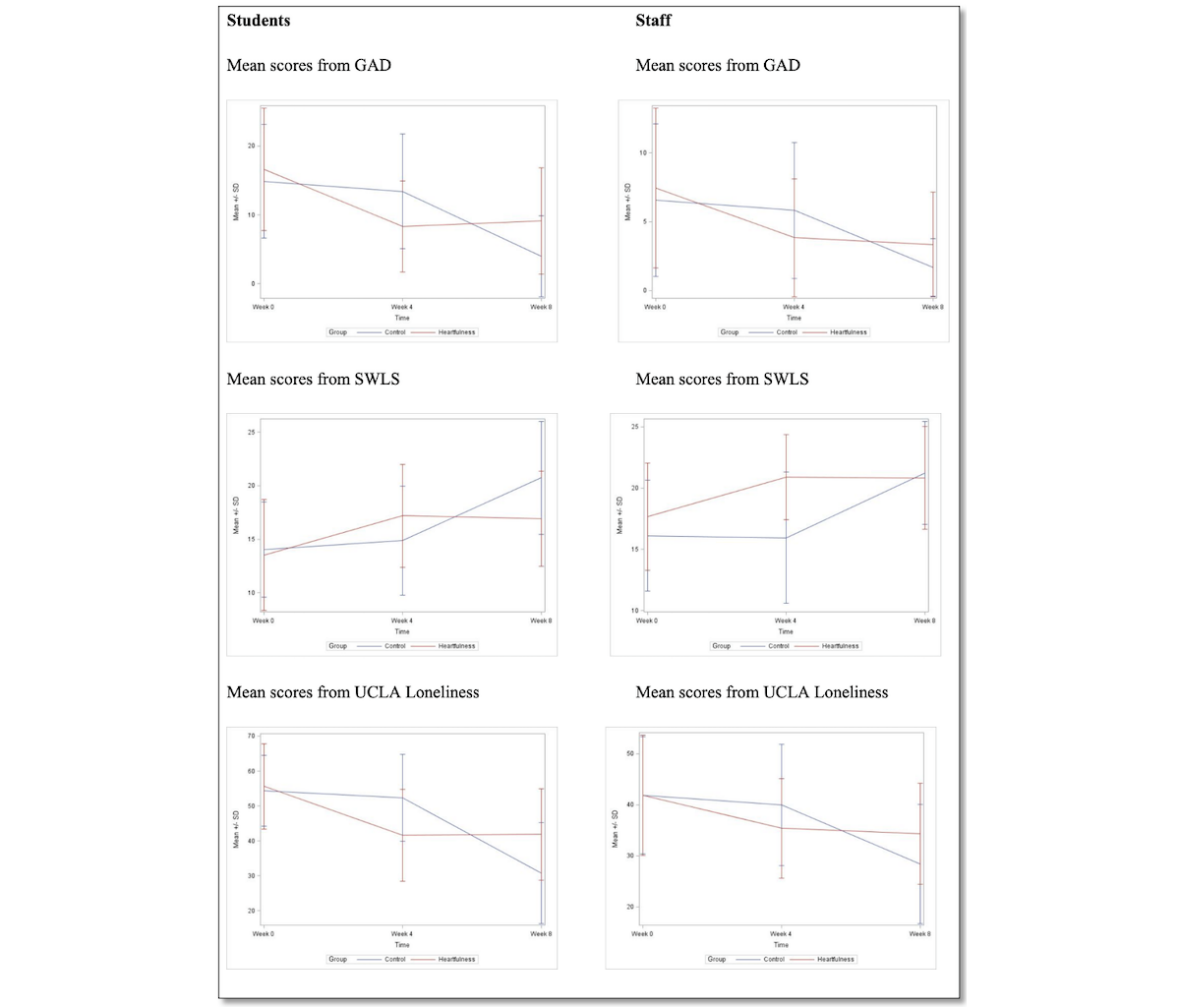
Mean scores for students and staff across time by group. GAD: generalized anxiety disorder; SWLS: Satisfaction With Life Scale; UCLA: University of California, Los Angeles.

The overall results of the Bonferroni sequentially rejective multiple comparison procedure for the students’ anxiety, satisfaction with life, and loneliness from the 3 scores are presented in Figure 3. Figure 3 also presents a visual of how all students did for the schools together. In the Heartfulness group, there was strong evidence to suggest a significant mean difference in GAD-7 scores between week 0 and week 4 at school 1 (*P*<.001; estimated mean difference was 11.06 points lower at week 4; 95% CI 5.26-16.86), at school 2 (*P*<.001; estimated mean difference was 11.25 points lower at week 4; 95% CI 4.55-17.95), and at school 3 (*P*=.03 estimated mean difference was 4.92 points lower at week 4; 95% CI 0.18-9.65). There was also strong evidence to suggest a significant mean difference in SWLS between week 0 and week 4 at school 1 (*P*<.001; estimated mean difference was 5.19 points higher at week 4; 95% CI –8.23 to –2.14), and at school 2 (*P*<.01); estimated mean difference was 4.92 points higher at week 4; 95% CI –8.43 to –1.40). Finally, there was strong evidence to suggest a significant mean difference in UCLA between week 0 and week 4 at school 1 (*P*<.001); estimated mean difference was 21.19 points lower at week 4; 95% CI 12-30.37), at School 2 (*P*<.01; estimated mean difference was 13 points higher at week 4; 95% CI 2.39-23.61), and at school 3 (*P*<.01; estimated mean difference was 9.71 points lower at week 4; 95% CI 2.21-17.21). See Table S1 in Multimedia Appendix 3 and Multimedia Appendix 4 for details on the effects of intervention on students.

### Intervention Effects on Staff

The overall results of the Bonferroni sequentially rejective multiple comparison procedure for the group×time interaction from the 3 scores are presented in Figure 3 presents a visual of how all staff did for the schools together. In the Heartfulness group, there was strong evidence to suggest a significant mean difference in GAD-7 scores between week 0 and week 4 at all schools (*P*<.001; estimated mean difference was 3.61 points lower at week 4; 95% CI 1.68-5.54). There was also strong evidence to suggest a significant mean difference in SWLS between week 0 and week 4 at school 2 (*P*<.001; estimated mean difference was 5.73 points higher at week 4; 95% CI –7.95 to –3.51). Finally, there was strong evidence to suggest a significant mean difference in UCLA between week 0 and week 4 at all schools (*P*; estimated mean difference was 6.44 points lower at week 4; 95% CI 1.74-11.14). See Table S1 in Multimedia Appendix 5 and Multimedia Appendix 6 for details on the effects of intervention on students.

## Discussion

### Principal Findings

This study explored the impact of a 4-week self-care program on the students and staff in 3 high schools with varying settings and backgrounds. The main findings indicate that the Heartfulness group exhibited a significant decrease in anxiety and loneliness scores across all 3 schools, alongside an improvement in satisfaction with life scores from week 0 to week 4. Conversely, the control group, which did not engage in the program during the first 4 weeks, showed similar improvements in these measures from week 4 to week 8, aligning with their participation in the program during this latter period. The inclusion of experimental and control groups is significant as it provides a robust framework for assessing the efficacy of the intervention. By comparing the Heartfulness group to the control group, which initially did not receive the intervention, the study ensures that observed changes in mental health outcomes can be attributed to the program rather than other external factors. This design enhances the credibility and validity of the findings, highlighting the direct impact of the Heartfulness Self-Care program on reducing anxiety and loneliness while improving life satisfaction.

The mental health consequences of COVID-19 have been well-documented, with numerous studies predicting a global mental health crisis exacerbated by the pandemic, especially among vulnerable populations [36]. Country-specific data have revealed high levels of stress, depression and anxiety symptoms, increased loneliness, and suicidal ideation [37-41]. The implementation of digital interventions in different settings is usually well received and particularly engaging for adolescents as well as the staff [42]. This study’s findings align with previous research, suggesting that the Heartfulness Self-Care program is effective in reducing anxiety and loneliness and improving life satisfaction among participants.

The pandemic induced severe educational and social changes, including social distancing and modifications in learning environments, which triggered a decline in mental health in schools. New COVID-19 protocols increased anxiety among both students and staff, and the return to school posed significant challenges. In addition, many individuals’ preexisting mental health conditions deteriorated due to a lack of access to appropriate services [43]. A school community needs to invest in developing a sense of connectedness in young adults and address social-emotional building to prepare them for their future. The Heartfulness Self-Care program provided a positive intervention that offered support to the students and staff after a difficult period of quarantine and lockdown. This program provided an opportunity for the students and staff to build specific skills, such as reflection, observation, positivity, time management, and goal setting, to handle anxiety and loneliness, and derive satisfaction with life. Supporting the whole school community is critical to prevent significant adverse consequences for students, and the school staff. The findings from this study can be used to alleviate the mental health burden and enhance mental wellness in the postpandemic era.

This study’s theoretical contribution lies in its emphasis on the entire school community, which includes both students and staff. While many studies may focus solely on students, this research recognizes that the mental health of educators and school personnel is equally important. It underscores the interconnectedness of the school community and how the well-being of 1 group can impact the other. This approach broadens the perspective on mental health interventions within an educational setting. It recognizes that the mental health consequences of the pandemic are not limited to a single aspect but encompass a wide range of psychological and emotional states, including anxiety, loneliness, and life satisfaction. By exploring the impact of the Heartfulness Self-Care program on these various aspects, the study acknowledges the complexity of mental well-being during a crisis. Methodologically, the study’s longitudinal design, with assessments at multiple time points (weeks 0, 4, and 8), allows for a thorough understanding of how mental health indicators evolve over time and how interventions can have lasting effects. Conducting the study in real-world educational settings with participants from diverse backgrounds enhances the ecological validity of the findings, reflecting the varied experiences and challenges faced by schools and students.

These contributions collectively advance our understanding of addressing the mental health consequences of a global crisis like the COVID-19 pandemic, offering valuable insights for both theory and practice in education and psychology. By integrating comparisons with previous studies and providing a detailed analysis of the results, this discussion underscores the effectiveness of the Heartfulness Self-Care program and its potential as a scalable intervention to support mental health in school communities.

### Limitations

The results of the study revealed strong evidence on the impact of the self-care program on anxiety, satisfaction with life, and loneliness in students and staff, however, limitations need to be considered when the study results are interpreted. First, although the study population seemingly is representative of 3 different schools and locations, the sample size limits the generalizability of the findings. Given the favorable results, a replication study with a larger sample may be useful. Second, the self-care program was offered during the second semester in the United States and students’ year-end examination, which was held in mid-March in India. Due to the change in exam pattern caused by the pandemic, students and staff faced issues with managing time to consistently attend the webinars for the program. If the self-care program had been held at another time, more students would have partaken willingly and enthusiastically. Finally, we measured anxiety, satisfaction with life, and loneliness with 3 quantitative self-rating scales. Future studies might include clinical and qualitative assessments of anxiety, life satisfaction, and loneliness to go in-depth in exploring the impact of this program.

Practical Implications

The implications of this study are substantial for educators, school psychologists, and counselors. Incorporating the findings into practice can help create a more supportive and nurturing learning environment. Here are some practical implications:

1. Integrating self-care programs: schools should consider integrating self-care programs into the curriculum or making them readily available to students and staff. These programs can include Heartfulness relaxation and meditation exercises, stress management techniques, and strategies for building emotional resilience.
2. Mental health education: it is crucial to include mental health education as part of the school curriculum. This should cover topics like recognizing and managing stress, anxiety, and emotional loneliness.
3. Peer support: encouraging peer support programs and mentorship within the school community can foster a sense of belonging and reduce feelings of isolation. Older students or staff members can offer guidance and emotional support to those in need.
4. Professional development: provide teachers and school staff with training in recognizing signs of mental distress and how to support students and colleagues effectively. This can include workshops on active listening, self-care, and empathy.
5. Web-based resources: maintain a library of web-based resources that students and staff can access to learn more about self-care, mental health, and emotional well-being.

Future Research Avenues

The authors recommend the following avenues of research to build upon the findings of this study:

1. Long-term effects: conduct longitudinal studies to assess the long-term effects of these web-based interventions on the mental health and well-being of students and staff, even after the pandemic has subsided.
2. Comparative studies: compare the effectiveness of different self-care tools and interventions to determine which approaches are most beneficial for different demographics within the school community.
3. Cultural sensitivity: explore the cultural nuances of mental health within school communities and adapt interventions to be culturally sensitive and effective.
4. Additional variables: future investigations should consider including additional variables such as academic performance, attendance rates, and the impact on the family and social life of students and staff.
5. Effectiveness of peer support: investigate the effectiveness of peer support and mentorship programs in promoting mental health within the school community.

By further exploring these research avenues, we can develop a comprehensive understanding of how to use digital interventions to best support the mental health and well-being of high school students and staff, thereby promoting a positive and nurturing learning environment.

### Conclusion

The COVID-19 pandemic has profoundly impacted every aspect of our lives, especially within the school community. Students and staff have had to adapt to new ways of attending school, navigating hybrid classes, and following social distancing measures. These changes have limited classroom experiences and social interactions, leading to increased fear, anxiety, and loneliness. Addressing the mental health of teens and adults in the school community, both during and after the pandemic, is now urgently needed.

This study has uniquely addressed a multitude of challenges stemming from the pandemic, by focusing on the well-being and safety of the school community. It is evident from our findings that the digital intervention examined in this study, using self-care tools, has effectively reduced anxiety and loneliness while improving overall life satisfaction. These results highlight the need for more preventive, accessible, and noninvasive programs like the one used in this study. In addition, these findings emphasize the importance of further research on the mental health of high school students and staff, using heart-based nurturing tools to promote well-being within the school community.

## Appendix

Multimedia Appendix 1 Demographic data of students (N=101).

Multimedia Appendix 2 Demographic data of staff (N=78).

Multimedia Appendix 3 Comparison results for GAD, SWLS and UCLA scores in students. GAD: generalized anxiety disorder; SWLS: Satisfaction With Life Scale; UCLA: University of California, Los Angeles.

Multimedia Appendix 4 Intervention effects on students.

Multimedia Appendix 5 Comparison results for GAD, SWLS and UCLA scores in staff. GAD: generalized anxiety disorder; SWLS: Satisfaction With Life Scale; UCLA: University of California, Los Angeles.

Multimedia Appendix 6 Intervention effects on staff.

Multimedia Appendix 7 CONSORT-EHEALTH (V 1.6.1) checklist.

## Abbreviations

CONSORT: Consolidated Standards of Reporting Trials
GAD-7: Generalized Anxiety Disorder-7 Scale
SWLS: Satisfaction With Life Scale
SWLS-C: Satisfaction With Life Scale-Child
UCLA: University of California, Los Angeles

## Data Availability

The data set generated and analyzed during the current study is available from Iyer et al [44].
