# Supplementary material for: Self-Care Program as a Tool for Alleviating Anxiety and Loneliness and Promoting Satisfaction With Life in High School Students and Staff: Randomized Survey Study
Source: JMIR Form Res. 2024 Sep 30;8:e56355. doi: 10.2196/56355 (PMC11474114; doi:10.2196/56355)
Supplement: Multimedia Appendix 1 [file formative_v8i1e56355_app1.docx]

**Table S1**

| *Variable* | *Frequency* | *Percent* |
| --- | --- | --- |
| **Gender** |  |  |
| Female | 77 | 76.24 |
| Male | 19 | 18.81 |
| Prefer not to say | 5 | 4.95 |
| **Groups** |  |  |
| Control | 49 | 48.51 |
| Heartfulness | 52 | 51.49 |
| **Grade Levels** |  |  |
| 9 | 11 | 10.89 |
| 10 | 23 | 22.77 |
| 11 | 44 | 43.56 |
| 12 | 23 | 22.77 |
