# Supplementary material for: Self-Care Program as a Tool for Alleviating Anxiety and Loneliness and Promoting Satisfaction With Life in High School Students and Staff: Randomized Survey Study
Source: JMIR Form Res. 2024 Sep 30;8:e56355. doi: 10.2196/56355 (PMC11474114; doi:10.2196/56355)
Supplement: Multimedia Appendix 2 [file formative_v8i1e56355_app2.docx]

**Table S1**

| *Variable* | *Frequency* | *Percent* |
| --- | --- | --- |
| **Gender** |  |  |
| F | 62 | 79.49 |
| M | 16 | 20.51 |
| **Groups** |  |  |
| Control | 37 | 47.44 |
| Heartfulness | 41 | 52.56 |
| **Profession** |  |  |
| Admin | 7 | 8.97 |
| Coach | 1 | 1.28 |
| Counselor | 3 | 3.85 |
| Librarian | 1 | 1.28 |
| Media Center Paraprofessional | 1 | 1.28 |
| Media Paraprofessional | 1 | 1.28 |
| Paraprofessional | 1 | 1.28 |
| Social Worker | 1 | 1.28 |
| Special Education Aides | 1 | 1.28 |
| Success Coach | 5 | 6.41 |
| Teacher | 55 | 70.51 |
| Warden | 1 | 1.28 |
