# Supplementary material for: Self-Care Program as a Tool for Alleviating Anxiety and Loneliness and Promoting Satisfaction With Life in High School Students and Staff: Randomized Survey Study
Source: JMIR Form Res. 2024 Sep 30;8:e56355. doi: 10.2196/56355 (PMC11474114; doi:10.2196/56355)
Supplement: Multimedia Appendix 3 [file formative_v8i1e56355_app3.docx]

**Table S1**

| Comparison | Estimate | p-value | 95% C.I. |
| --- | --- | --- | --- |
| **GAD measuring Anxiety** |  |  |  |
| School 1: Control Week 0 - Heartfulness Week 0 | -4.27 | .99 | (-12.98, 4.44) |
| School 1: Heartfulness Week 0 - Heartfulness Week 4 | 11.06 | .000** | (5.26, 16.86) |
| School 1: Heartfulness Week 4 - Heartfulness Week 8 | -0.81 | .99 | (-6.61, 4.99) |
| School 1: Heartfulness Week 0 - Heartfulness Week 8 | 10.25 | .000** | (3.06, 17.44) |
| School 1: Control Week 0 - Control Week 4 | 0.73 | .99 | (-5.26, 6.72) |
| School 1: Control Week 4 - Control Week 8 | 5.40 | .1 | (-0.59, 11.39) |
| School 1: Control Week 0 - Control Week 8 | 6.13 | .19 | (-1.29, 13.55) |
| School 2: Control Week 0 - Heartfulness Week 0 | 0.25 | .99 | (-9.64, 10.14) |
| School 2: Heartfulness Week 0 - Heartfulness Week 4 | 11.25 | .000** | (4.55, 17.95) |
| School 2: Heartfulness Week 4 - Heartfulness Week 8 | -0.58 | .99 | (-7.28, 6.11) |
| School 2: Heartfulness Week 0 - Heartfulness Week 8 | 10.67 | .000** | (2.37, 18.96) |
| School 2: Control Week 0 - Control Week 4 | 0.00 | .99 | (-6.7, 6.7) |
| School 2: Control Week 4 - Control Week 8 | 20.50 | .000** | (13.8, 27.2) |
| School 2: Control Week 0 - Control Week 8 | 20.50 | .000** | (12.2, 28.8) |
| School 3: Control Week 0 - Heartfulness Week 0 | -1.30 | .99 | (-8.45, 5.86) |
| School 3: Heartfulness Week 0 - Heartfulness Week 4 | 4.92 | .025* | (0.18, 9.65) |
| School 3: Heartfulness Week 4 - Heartfulness Week 8 | -0.88 | .99 | (-5.61, 3.86) |
| School 3: Heartfulness Week 0 - Heartfulness Week 8 | 4.04 | .6 | (-1.83, 9.91) |
| School 3: Control Week 0 - Control Week 4 | 2.77 | .99 | (-2.17, 7.72) |
| School 3: Control Week 4 - Control Week 8 | 6.09 | .003* | (1.14, 11.04) |
| School 3: Control Week 0 - Control Week 8 | 8.86 | .000** | (2.74, 14.99) |
| **SWLS measuring Satisfaction with Life** |  |  |  |
| School 1: Control Week 0 - Heartfulness Week 0 | -0.09 | .99 | (-5.63, 5.46) |
| School 1: Heartfulness Week 0 - Heartfulness Week 4 | -5.19 | .000** | (-8.23, -2.14) |
| School 1: Heartfulness Week 4 - Heartfulness Week 8 | 0.25 | .99 | (-2.8, 3.3) |
| School 1: Heartfulness Week 0 - Heartfulness Week 8 | -4.94 | .002* | (-8.89, -0.99) |
| School 1: Control Week 0 - Control Week 4 | 0.27 | .99 | (-2.88, 3.41) |
| School 1: Control Week 4 - Control Week 8 | -2.60 | .21 | (-5.75, 0.55) |
| School 1: Control Week 0 - Control Week 8 | -2.33 | .99 | (-6.41, 1.74) |
| School 2: Control Week 0 - Heartfulness Week 0 | 1.50 | .99 | (-4.8, 7.8) |
| School 2: Heartfulness Week 0 - Heartfulness Week 4 | -4.92 | .000** | (-8.43, -1.4) |
| School 2: Heartfulness 4 - Heartfulness Week 8 | -0.25 | .99 | (-3.77, 3.27) |
| School 2: Heartfulness Week 0 - Heartfulness Week 8 | -5.17 | .008* | (-9.73, -0.61) |
| School 2: Control Week 0 - Control Week 4 | -0.50 | .99 | (-4.02, 3.02) |
| School 2: Control Week 4 - Control Week 8 | -10.33 | .000** | (-13.85, -6.82) |
| School 2: Control Week 0 - Control Week 8 | -10.83 | .000** | (-15.39, -6.27) |
| School 3: Control Week 0 - Heartfulness Week 0 | 0.44 | .99 | (-4.12, 4.99) |
| School 3: Heartfulness Week 0 - Heartfulness Week 4 | -2.04 | .21 | (-4.53, 0.45) |
| School 3: Heartfulness Week 4 - Heartfulness Week 8 | 0.58 | .99 | (-1.9, 3.07) |
| School 3: Heartfulness Week 0 - Heartfulness Week 8 | -1.46 | .99 | (-4.68, 1.77) |
| School 3: Control Week 0 - Control Week 4 | -1.82 | .6 | (-4.42, 0.78) |
| School 3: Control Week 4 - Control Week 8 | -5.64 | .000** | (-8.23, -3.04) |
| School 3: Control Week 0 - Control Week 8 | -7.45 | .000** | (-10.82, -4.09) |
| **UCLA Loneliness measuring Loneliness** |  |  |  |
| School 1: Control Week 0 - Heartfulness Week 0 | -4.16 | .99 | (-18.33, 10.01) |
| School 1: Heartfulness Week 0 - Heartfulness Week 4 | 21.19 | .000** | (12, 30.37) |
| School 1: Heartfulness Week 4 - Heartfulness Week 8 | 0.19 | .99 | (-9, 9.37) |
| School 1: Heartfulness Week 0 - Heartfulness Week 8 | 21.38 | .000** | (9.91, 32.84) |
| School 1: Control Week 0 - Control Week 4 | 0.13 | .99 | (-9.35, 9.62) |
| School 1: Control Week 4 - Control Week 8 | 10.20 | .015* | (0.71, 19.69) |
| School 1: Control Week 0 - Control Week 8 | 10.33 | .13 | (-1.51, 22.18) |
| School 2: Control Week 0 - Heartfulness Week 0 | -2.75 | .99 | (-18.85, 13.35) |
| School 2: Heartfulness Week 0 - Heartfulness Week 4 | 13.00 | .003* | (2.39, 23.61) |
| School 2: Heartfulness 4 - Heartfulness Week 8 | 2.00 | .99 | (-8.61, 12.61) |
| School 2: Heartfulness Week 0 - Heartfulness Week 8 | 15.00 | .008* | (1.76, 28.24) |
| School 2: Control Week 0 - Control Week 4 | 0.08 | .99 | (-10.52, 10.69) |
| School 2: Control Week 4 - Control Week 8 | 36.33 | .000** | (25.73, 46.94) |
| School 2: Control Week 0 - Control Week 8 | 36.42 | .000** | (23.17, 49.66) |
| School 3: Control Week 0 - Heartfulness Week 0 | 1.35 | .99 | (-10.29, 12.99) |
| School 3: Heartfulness Week 0 - Heartfulness Week 4 | 9.71 | .001* | (2.21, 17.21) |
| School 3: Heartfulness Week 4 - Heartfulness Week 8 | -1.58 | .99 | (-9.08, 5.92) |
| School 3: Heartfulness Week 0 - Heartfulness Week 8 | 8.13 | .13 | (-1.24, 17.49) |
| School 3: Control Week 0 - Control Week 4 | 4.55 | .99 | (-3.29, 12.38) |
| School 3: Control Week 4 - Control Week 8 | 21.18 | .000** | (13.35, 29.02) |
| School 3: Control Week 0 - Control Week 8 | 25.73 | .000** | (15.95, 35.51) |

*GAD* Generalized Anxiety Disorder, *SWLS* Satisfaction With Life Scale
