# Supplementary material for: Self-Care Program as a Tool for Alleviating Anxiety and Loneliness and Promoting Satisfaction With Life in High School Students and Staff: Randomized Survey Study
Source: JMIR Form Res. 2024 Sep 30;8:e56355. doi: 10.2196/56355 (PMC11474114; doi:10.2196/56355)
Supplement: Multimedia Appendix 4 [file formative_v8i1e56355_app4.docx]

The overall results of the Bonferroni sequentially rejective multiple comparison procedure for the students’ anxiety, satisfaction with life and loneliness from the three scores are presented in Figure 3 presents a visual of how all students did for the schools together.

Anxiety

In the Heartfulness group, there was strong evidence to suggest a significant mean difference in GAD between Week 0 and Week 4 at School 1 (p<.0001; estimated mean difference was 11.06 points lower at Week 4; 95% CI of (5.26, 16.86)), at School 2 (p< 0.0001; estimated mean difference was 11.25 points lower at Week 4; 95% CI of (4.55, 17.95)), and at School 3 (p =.0253; estimated mean difference was 4.92 points lower at Week 4; 95% Confidence Interval (CI) of (0.18, 9.65)). There was also strong evidence to suggest a significant mean difference in GAD between Week 0 and Week 8 at School 1 (p =.0002; estimated mean difference was 10.25 points lower at Week 8; 95% CI of (3.06, 17.44)), and at School 2 (p =.0012; estimated mean difference was 10.67 points lower at Week 8; 95% CI of (2.37, 18.96)).

In the control group, there was strong evidence to suggest a significant mean difference in GAD between Week 4 and Week 8 at School 2 (p<.0001; estimated mean difference of 20.5 points lower at Week 8; 95% CI of (13.8, 27.2)), and at School 3 (p=.0025; estimated mean difference of 6.09 points lower at Week 8; 95% CI of (1.14, 11.04). There was also strong evidence to suggest a significant mean difference in GAD between Week 0 and Week 8 at School 2 (p<.0001; estimated mean difference was 20.5 points lower at Week 8; 95% CI of (12.2, 28.8)), and at School 3 (p = 0.0001; the estimated mean difference was 8.86 points lower at Week 8; 95% CI of (2.74, 14.99)).

Satisfaction with Life

In the Heartfulness group, there was strong evidence to suggest a significant mean difference in SWLS between Week 0 and Week 4 at School 1 (p<.0001; estimated mean difference was 5.19 points higher at Week 4; 95% CI of (-8.23, -2.14)), and at School 2 (p=.0003); estimated mean difference was 4.92 points higher at Week 4; 95% CI of (-8.43, -1.40)). There was also strong evidence to suggest a significant mean difference in SWLS between Week 0 and Week 8 at School 1 (p=.0019; the estimated mean difference was 4.94 points lower at Week 8; 95% CI of (-8.89, -0.99)), and at School 2 (p=.008; estimated mean difference was 5.17 points higher at Week 8; 95% CI of (-9.73, - 0.61)).

In the Control group, there was strong evidence to suggest a significant mean difference in SWLS between Week 4 and Week 8 at School 2 (p<.0001; the estimated mean difference was 10.33 points higher at Week 8; 95% CI of (-13.85, -6.82)), and at School 3 (p<.0001; the estimated mean difference was 5.64 points higher at Week 8; 95% CI of (-8.23, -3.04)). There was also strong evidence to suggest a significant mean difference in SWLS between Week 0 and Week 8 at School 2 (p<.0001; estimated mean difference was 10.83 points higher at Week 8; 95% CI of (-15.39, -6.27)), and at School 3 (p<.0001; the estimated mean difference was 7.45 points higher at Week 8; 95% CI of (-10.82, -4.09)).

Loneliness

In the Heartfulness group, there was strong evidence to suggest a significant mean difference in UCLA between Week 0 and Week 4 at School 1 (p<.0001); estimated mean difference was 21.19 points lower at Week 4; 95% CI of (12, 30.37)), at School 2 (p=.0025; estimated mean difference was 13 points higher at Week 4; 95% CI of (2.39, 23.61)), and at School 3 (p=.0011; estimated mean difference was 9.71 points lower at Week 4; 95% CI of (2.21, 17.21)). There was also strong evidence to suggest a significant mean difference in UCLA between Week 0 and Week 8 at School 1 (p<.0001; estimated mean difference was 21.38 points lower at Week 8; 95% CI of (9.91, 32.84)), and at School 2 (p=.0076; estimated mean difference was 15 points higher at Week 8; 95% CI of (1.76, 28.24)).

In the control group, there was strong evidence to suggest a significant mean difference in UCLA between Week 4 and Week 8 at School 1 (p=.0015; estimated mean difference was 10.2 points lower at Week 8; 95% CI of (0.71, 19.69)), at School 2 (p<.0001); estimated mean difference was 36.33 points lower at Week 8; 95% CI of (25.73, 46.94)), and at School 3 (p<.0001; estimated mean difference is 21.18 points lower at Week 8; 95% CI of (13.35, 29.02)). There was also strong evidence to suggest a significant mean difference in UCLA between Week 0 and Control Week 8 at School 2 (p<.0001; estimated mean difference was 36.42 points lower at Week 8; 95% CI of (23.17, 49.66)), and at School 3 (p<.0001); estimated mean difference was 25.73 points lower at Week 8; 95% CI of (15.95, 35.51)).
