# Supplementary material for: Self-Care Program as a Tool for Alleviating Anxiety and Loneliness and Promoting Satisfaction With Life in High School Students and Staff: Randomized Survey Study
Source: JMIR Form Res. 2024 Sep 30;8:e56355. doi: 10.2196/56355 (PMC11474114; doi:10.2196/56355)
Supplement: Multimedia Appendix 5 [file formative_v8i1e56355_app5.docx]

**Table S1**

| Comparison | Estimate | p-value | 95% C.I. |
| --- | --- | --- | --- |
| **GAD measuring Anxiety** |  |  |  |
| Week 0: Control - Heartfulness | -0.95 | .89 | (-3.75, 1.86) |
| Heartfulness: Week 0- Week 4 | 3.61 | .000** | (1.68, 5.54) |
| Heartfulness: Week 4 - Week 8 | 0.49 | .89 | (-1.44, 2.42) |
| Heartfulness: Week 0 - Week 8 | 4.10 | .000** | (1.74, 6.46) |
| Control: Week 0 - Week 4 | 0.76 | .89 | (-1.27, 2.79) |
| Control: Week 4 - Week 8 | 4.14 | .000** | (2.11, 6.16) |
| Control: Week 0 - Week 8 | 4.89 | .000** | (2.41, 7.37) |
| **SWLS measuring Satisfaction with Life** |  |  |  |
| School 1: Control Week 0 - Heartfulness Week 0 | -5.21 | .95 | (-9.88, -0.55) |
| School 1: Heartfulness Week 0 - Heartfulness Week 4 | -3.29 | .99 | (-6.54, -0.04) |
| School 1: Heartfulness Week 4 - Heartfulness Week 8 | -0.29 | .99 | (-3.54, 2.96) |
| School 1: Heartfulness Week 0 - Heartfulness Week 8 | -3.57 | .99 | (-7.52, 0.38) |
| School 1: Control Week 0 - Control Week 4 | 3.33 | .99 | (-0.18, 6.84) |
| School 1: Control Week 4 - Control Week 8 | -12.83 | .000** | (-16.34, -9.32) |
| School 1: Control Week 0 - Control Week 8 | -9.50 | .001* | (-13.77, -5.23) |
| School 2: Control Week 0 - Heartfulness Week 0 | -0.07 | .99 | (-3.13, 3) |
| School 2: Heartfulness Week 0 - Heartfulness Week 4 | -5.73 | .000** | (-7.95, -3.51) |
| School 2: Heartfulness 4 - Heartfulness Week 8 | 0.40 | .99 | (-1.82, 2.62) |
| School 2: Heartfulness Week 0 - Heartfulness Week 8 | -5.33 | .005* | (-8.03, -2.64) |
| School 2: Control Week 0 - Control Week 4 | -0.87 | .99 | (-3.09, 1.35) |
| School 2: Control Week 4 - Control Week 8 | -3.60 | .06 | (-5.82, -1.38) |
| School 2: Control Week 0 - Control Week 8 | -4.47 | .049* | (-7.16, -1.77) |
| School 3: Control Week 0 - Heartfulness Week 0 | -1.42 | .99 | (-4.26, 1.43) |
| School 3: Heartfulness Week 0 - Heartfulness Week 4 | -1.26 | .99 | (-3.24, 0.71) |
| School 3: Heartfulness 4 - Heartfulness Week 8 | -0.05 | .99 | (-2.03, 1.92) |
| School 3: Heartfulness Week 0 - Heartfulness Week 8 | -1.32 | .99 | (-3.71, 1.08) |
| School 3: Control Week 0 - Control Week 4 | -0.06 | .99 | (-2.21, 2.09) |
| School 3: Control Week 4 - Control Week 8 | -4.06 | .011* | (-6.21, -1.91) |
| School 3: Control Week 0 - Control Week 8 | -4.13 | .07 | (-6.74, -1.51) |
| **UCLA Loneliness measuring Loneliness** |  |  |  |
| Week 0: Control- Heartfulness | 0.20 | .99 | (-6.78, 7.17) |
| Heartfulness Week 0 - Week 4 | 6.44 | .001** | (1.74, 11.14) |
| Heartfulness: Week 4 - Week 8 | 1.02 | .99 | (-3.68, 5.73) |
| Heartfulness: Week 0 - Week 8 | 7.46 | .002* | (1.67, 13.25) |
| Control: Week 0 - Week 4 | 1.86 | .87 | (-3.08, 6.81) |
| Control: Week 4 - Week 8 | 11.57 | .000** | (6.62, 16.52) |
| Control: Week 0 - Week 8 | 13.43 | .000** | (7.34, 19.53) |

*GAD* Generalized Anxiety Disorder, *SWLS* Satisfaction With Life Scale
