# Supplementary material for: Self-Care Program as a Tool for Alleviating Anxiety and Loneliness and Promoting Satisfaction With Life in High School Students and Staff: Randomized Survey Study
Source: JMIR Form Res. 2024 Sep 30;8:e56355. doi: 10.2196/56355 (PMC11474114; doi:10.2196/56355)
Supplement: Multimedia Appendix 6 [file formative_v8i1e56355_app6.docx]

The overall results of the Bonferroni sequentially rejective multiple comparison procedure for the Group*Time interaction from the three scores are presented in Figure 3 presents a visual of how all staff did for the schools together.

Anxiety

In the Heartfulness group, there was strong evidence to suggest a significant mean difference in GAD between Week 0 and Week 4 at all schools (p<.0001; estimated mean difference was 3.61 points lower at Week 4; 95% CI of (1.68, 5.54)). There was also strong evidence to suggest a significant mean difference in GAD between Week 0 and Week 8 at all schools (p<.0001; estimated mean difference was 4.1 points lower at Week 8; 95% CI of (1.74, 6.46)).

In the control group, there was strong evidence to suggest a significant mean difference in GAD between Week 4 and Week 8 for all schools (p<.0001; estimated mean difference was 4.14 points lower at Week 8; 95% CI of (2.11, 6.16)). There was also strong evidence to suggest a significant mean difference in GAD between Week 0 and Week 8 at all schools (p<.0001; estimated mean difference was 4.89 points lower at Week 8; 95% CI of (2.41, 7.37)). Satisfaction with Life

In the Heartfulness group, there was strong evidence to suggest a significant mean difference in SWLS between Week 0 and Week 4 at School 2 (p<.0001; estimated mean difference was 5.73 points higher at Week 4; 95% CI of (-7.95, -3.51)). There was also strong evidence to suggest a significant mean difference in SWLS between Week 0 and Week 8 at School 2 (p=.0054; estimated mean difference was 5.33 points higher at Week 8; 95% CI of (-8.03, -2.64)).

In the control group, there was strong evidence to suggest a significant mean difference in SWLS between Week 4 and Week 8 at School 1 (p<.0001); estimated mean difference was 12.83 points higher at Week 8; 95% CI of (-16.34, -9.32)), and at School 3 (p=.011; estimated mean difference was 4.06 points higher at Week 8; 95% CI of (-6.21, -1.91)). There was also strong evidence to suggest a significant mean difference in SWLS between Week 0 and Week 8 at School 1 (p=.0008; estimated mean difference was 9.5 points higher at Week 8; 95% CI of (-13.77, -5.23)), and at School 2 (p=.049; estimated mean difference was 4.47 points higher at Week 8; 95% CI of (-7.16, -1.77)).

Loneliness

In the Heartfulness group, there was strong evidence to suggest a significant mean difference in UCLA between Week 0 and Week 4 at all schools (p=.0012; estimated mean difference was 6.44 points lower at Week 4; 95% CI of (1.74, 11.14)). There was also strong evidence to suggest a significant mean difference in UCLA between Week 0 and Week 8 at all schools (p=.0023; estimated mean difference was 7.46 points lower at Week 8; 95% CI of (1.67, 13.25)).

In the control group, there was strong evidence to suggest a significant mean difference in UCLA between Week 4 and Week 8 for all schools (p<.0001; estimated mean difference was 11.57 points lower at Week 8; 95% CI of (6.62, 16.52)). There was also strong evidence to suggest a significant mean difference in UCLA between Week 0 and Week 8 at all schools (p<.0001; estimated mean difference was 13.43 points lower at Week 8; 95% CI of (7.34, 19.53)).
